# Conserved Residues in the C-Terminal Domain Affect the Structure and Function of CYP38 in Arabidopsis

**DOI:** 10.3389/fpls.2021.630644

**Published:** 2021-02-25

**Authors:** Lujing Shi, Lele Du, Jingru Wen, Xiumei Zong, Wene Zhao, Juan Wang, Min Xu, Yuhua Wang, Aigen Fu

**Affiliations:** Chinese Education Ministry’s Key Laboratory of Western Resources and Modern Biotechnology, Key Laboratory of Biotechnology Shaanxi Province, College of Life Sciences, Northwest University, Xi’an, China

**Keywords:** PSII assembly and repair, site directed mutagenesis, configuration change, intramolecular interaction, the C-terminal domain, CYP38

## Abstract

Arabidopsis cyclophilin38 (CYP38) is a thylakoid lumen protein critial for PSII assembly and maintenance, and its C-terminal region serves as the target binding domain. We hypothesized that four conserved residues (R290, F294, Q372, and F374) in the C-terminal domain are critical for the structure and function of CYP38. In yeast two-hybrid and protein pull-down assays, CYP38s with single-sited mutations (R290A, F294A, Q372A, or F374A) did not interact with the CP47 E-loop as the wild-type CYP38. In contrast, CYP38 with the R290A/F294A/Q372A/F374A quadruple mutation could bind the CP47 E-loop. Gene transformation analysis showed that the quadruple mutation prevented CYP38 to efficiently complement the mutant phenotype of *cyp38*. The C-terminal domain half protein with the quadruple mutation, like the wild-type one, could interact with the N-terminal domain or the CP47 E-loop *in vitro*. The *cyp38* plants expressing CYP38 with the quadruple mutation showed a similar BN-PAGE profile as *cyp38*, but distinct from the wild type. The CYP38 protein with the quadruple mutation associated with the thylakoid membrane less efficiently than the wild-type CYP38. We concluded that these four conserved residues are indispensable as changes of all these residues together resulted in a subtle conformational change of CYP38 and reduced its intramolecular N-C interaction and the ability to associate with the thylakoid membrane, thus impairing its function in chloroplast.

## Introduction

FK506 binding proteins (FKBPs) and cyclophilins (CYPs) are cellular receptors for immunosuppressant drugs FK506 and cyclosporine A, respectively (Harding et al., 1989; Siekierka et al., 1989; Standaert et al., 1990). They are collectively referred as immunophilins and share a common feature of peptidyl-prolyl cis/trans isomerase (PPIase) activity in spite of a lack of significant sequence similarity (Romano et al., 2005). PPIases catalyze the cis/trans conversion of X-prolyl peptide bonds, a rate-limiting step in protein folding (Fischer et al., 1989; Reimer and Fischer, 2002). Immunophilins are widely present from prokaryotic to eukaryotic organisms, and play critical roles in various essential biological processes (Shi and Fu, 2015). Notably, photosynthetic organisms harbor a remarkably large number of immunophilins. For instance, 49 members (23 FKBPs and 26 CYPs) were found in Chlamydomonas (Vallon, 2005); and 56 (29 FKBPs and 27 CYPs) and 52 (23 FKBPs and 29CYPs) were identified in rice (Ahn et al., 2010) and Arabidopsis (He et al., 2004), respectively. Immunophilins in photosynthetic organisms are functionally critical for photosynthesis, as well as for other important processes, including protein folding, development, and stress response (Luan, 1998; Buchanan and Luan, 2005).

In Arabidopsis, a large number of immunophilins are present in chloroplast: 1 CYP in the stroma, 5 CYPs and 11 FKBPs in the thylakoid lumen (He et al., 2004). The stroma localized CYP20-3/ROC4 was reported to function in the repair process of photosystem II (PSII), and CYP20-3 deficiency caused an increased sensitivity to high light stress in Arabidopsis (Cai et al., 2008). Arabidopsis CYP20-3 was also identified as an acceptor for jasmonates in chloroplast, and played a key role in cysteine synthesis and chloroplast redox homeostasis (Motohashi et al., 2003; Laxa et al., 2007; Dominguez-Solis et al., 2008; Park et al., 2013). FKBP13, a thylakoid lumen immunophilin, was found to interact with the Rieske subunit of Cytb6f complex and regulate its accumulation in Arabidopsis (Gupta et al., 2002; Gopalan et al., 2004; Gollan et al., 2011; Rochaix, 2011). FKBP16-1 was showed to be a high light-induced protein in Arabidopsis and could regulate the stability of PsaL, a subunit of photosystem I (PSI) (Seok et al., 2014). Arabidopsis FKBP16-2 is a peripheral subunit of NDH complex on the thylakoid lumen side, and participates in the assembly or/and maintenance of the NDH complex (Peng et al., 2009). An Arabidopsis mutant lacking FKBP20-2 exhibited a reduced growth, lower PSII activity, and reduced level of PSII supercomplex (PSII-SC), suggesting that FKBP20-2 plays a vital role in the assembly of PSII-SC (Lima et al., 2006). It was reported that CYP20-2 is also an auxiliary subunit of the NDH complex in the thylakoid lumen. However CYP20-2 appears to be not essential for NDH function, given that Arabidopsis mutant depleted of CYP20-2 grew as well as the wild type (Sirpiö et al., 2009). Interestingly, another report showed that Arabidopsis CYP20-2 could be directed into the nucleus and interact with BZR1, a transcription factor responding to brassinosteroids (BRs); the PPIase activity of CYP20-2 could mediate conformational change of BZR1, and further influence plant development (Zhang et al., 2013). In rice, CYP20-2 was found to be important for responding to environmental stresses (Kim et al., 2012). Very recently, rice CYP20-2 was reported to be dual-localized in chloroplasts and nucleus. While the nuclear variant interacts with SLR1, the chloroplast variant was shown to associate with FSD2 to integrate chilling tolerance and plant growth (Ge et al., 2020).

The PPIase activity of immunophilins was initially thought to be essential for their physiological roles, however, it was found that there is no tight correlation between the PPIase activity and their functions (Zydowsky et al., 1992; Etzkorn et al., 1994; Futer et al., 1995). In Arabidopsis, FKBP13 and CYP20-2 are two active PPIases in the thylakoid lumen, but inactivation of both FKBP13 and CYP20-2 does not obviously influence plant growth and development (Shapiguzov et al., 2006; Edvardsson et al., 2007; Ingelsson et al., 2009). The majority of chloroplast immunophilins have undergone substantial changes of conserved residues required for PPIase activity and became inactive PPIases during evolution; nevertheless, they could be still crucial for chloroplast functions (He et al., 2004; Buchanan and Luan, 2005).

CYP38, a thylakoid lumen immunophilin, gave rise to extensive studies due to a stunted growth phenotype and the high light sensitivity of the Arabidopsis *cyp38* mutant (Fu et al., 2007; Sirpiö et al., 2008). The severe defects in the PSII-SC in *cyp38* suggested that CYP38 plays a crucial role in the assembly and maintenance of PSII in Arabidopsis (Fu et al., 2007). Arabidopsis CYP38 was found to be responsible for the correct assembly of D1 and CP43 during PSII assembly and repair, and CYP38 deficiency affected the donor side of PSII, rendering *cyp38* plants more susceptible to photodamage (Sirpiö et al., 2008). In another report, CYP38 was shown to reduce D1 dephosphorylation and degradation by suppressing the GTPase activity of PsbO2 (Wang et al., 2015). In addition, CYP38 was found to be involved in maintaining chloroplast morphogenesis and thylakoid stacking in Arabidopsis (Vojta et al., 2019). The CYP38 ortholog in spinach, TLP40, was identified in an attempt to purify a chloroplast phosphatase, and was proposed to regulate PSII phosphorylation (Fulgosi et al., 1998; Vener et al., 1999; Rokka et al., 2000).

Arabidopsis CYP38 contains two distinct domains: an N-terminal α-helical bundle and a C-terminal β-sheet barrel. Crystal structure analysis showed that the N-terminal domain is closely packed together with the C-terminal domain through a strong intramolecular interaction. Consistently, *in vitro* assays showed that only the C-terminal domain, but neither the mature full length CYP38 nor its N-terminal domain, could interact with its potential target, the CP47 E-loop (Vasudevan et al., 2012). The C-terminal domain of CYP38 has a divergent CYP-type PPIase structure, and enzyme activity assays showed that CYP38 has no PPIase activity (Shapiguzov et al., 2006; Vasudevan et al., 2012). Active CYP-type PPIases contain several conserved key residues necessary for PPIase activity (Kallen et al., 1991; Zydowsky et al., 1992; Pflugl et al., 1993). Site directed mutagenesis analysis showed that seven conserved residues in the human CYPA are critical for its PPIase activity, but only four of those are conserved in Arabidopsis CYP38 (Edvardsson et al., 2007). It is likely that Arabidopsis CYP38 is a non-canonical CYP with a novel function rather than a PPIase.

We proposed that the C-terminal domain of CYP38 of Arabidopsis serves as the target binding domain, and its conserved residues (R290, F294, Q372, and F374) are important for the structure and function. To exam this possibility, we used a site directed mutagenesis approach to create alternations in these conserved residues. Then we tested impact of these changes on the structure of CYP38 *in vitro* via yeast two-hybrid and protein pull-down methods. We also examined the functional effects *in planta* via introducing mutated proteins into the Arabidopsis *cyp38* mutant. We found that these four conserved residues are required for the structure and function of CYP38, probably by maintaining the structural integrity and the putative chaperone-like activity of the C-terminal domain. Our results shed light on the working mechanism of CYP38 and they could provide a clue to study the structure and function of other immunophilins.

## Materials and Methods

### Primers

All the primers used in this study are listed in Supplementary Table 1.

### Plant Materials and Growth Conditions

Wild-type Arabidopsis, *cyp38* mutant (Garlic 206 38), and transgenic plants used in this study are all in the background of the Columbia ecotype. Generally, plants were grown on soil at 25°C under low light (30 μmol m^–2^ s^–1^) for 14 days, then subjected to continuous illumination (80–100 μmol m^–2^ s^–1^), similar to growth conditions described earlier (Fu et al., 2007). The transgenic plants carrying wild type and mutated *CYP38* were created as following. The full length coding sequences (CDSs) of wild type and mutated CYP38s were cloned behind the cauliflower mosaic virus (CaMV) 35S promoter in the binary vector pRI101-AN, and the constructs were transferred into *Agrobacterium tumefaciens* strain GV3101 and introduced into *cyp38* via by the floral dip method (Clough and Bent, 1998). T_1_ generation plants were grown and selected on 1/2 MS medium containing 25 μg mL^–1^ kanamycin, and then verified by PCR.

### Site Directed Mutagenesis

Basically, the procedure as described before was followed (Fu et al., 2005). The full length CDS of CYP38 was cloned into the vector pBluescript II KS (−). Site directed mutations were created in the *CYP38* coding region by PCR-based amplification using PrimeSTAR Max DNA Polymerase (Takara, Japan). The primers are listed in Supplementary Table 1. After 30 cycles of PCR amplification at 98°C for 15 s, 56°C for 15 s, and 72°C for 1 min in a thermal cycler, the products were digested with *Dpn*I (New England Biolabs, United States) for 2 h at 37°C, and 5 μL of the reaction was used to transform *E. coli* DH5a competent cells. The resulting vectors were confirmed by DNA sequence analysis.

### Preparation of Total Leaf Protein and Immunoblotting Analysis

Total leaf proteins were extracted by grinding leaf material and resuspended in 2x SDS loading buffer (125 mM Tris-HCl, pH 6.8, 4% (w/v) SDS, 2% (v/v) 2-mercaptoethanol, 0.001% (w/v) bromphenol blue, 20% (v/v) glycerol), followed by incubation at 98°C for 10 min. Immunoblotting was performed as before (Fu et al., 2005). Protein samples were separated by SDS-PAGE and transferred to nitrocellulose membranes for immunoblotting analysis. Chemiluminescent signals were generated using the ECL Plus reagent (Tanon, China) and visualized using the Tanon-5200 Chemiluminescent Imaging System (Tanon, China). Equal loading was monitored by staining membranes with Ponceau S (Sigma-Aldrich, United States).

### Yeast Two-Hybrid Analysis

The DNA sequences encoding wild type and mutated CYP38s were cloned into pGBKT7 and used as baits. The coding sequences of CP47 E-loop was cloned into pGADT7 and used as prey. Yeast transformants were selected on synthetic dropout medium lacking Leu and Trp (−LT). To screen protein–protein interactions, transformed yeast cells of different dilutions (1/10, 1/100, and 1/1,000) were spotted onto the selection media lacking Leu, Trp, Ade, and His (−LTAH), and the cell growth was used as an indicator of protein-protein interaction (Lee et al., 2007).

### *In vitro* Pull-Down Assay

The DNA sequences encoding wild type and mutated CYP38s were cloned downstream of the GST tag in the expression vector pGEX-4T-3, and *CP47 E-loop* cDNA was cloned downstream of the His tag in the expression vector pET-28a (+). All constructs were introduced into the *E. coli* BL21 (DE3) strain, and recombinant protein expression was induced with 0.1 mM isopropyl-β-D-thiogalactoside at 20°C for overnight. The GST fusion proteins and His fusion protein were purified through affinity chromatograph with Glutathione sepharose^TM^ 4B (GE Healthcare, United States) and Ni-NTA Agarose (QIAGEN, Germany) according to the manufacturer’s instruction, respectively. The *in vitro* pull-down assay was performed as previously described with minor modifications (Li et al., 2016), Ni-NTA Agarose containing 500 ng His-CP47 E-loop was incubated with 500 ng free GST, or GST-fused prey proteins in Pull-down buffer (140 mM NaCl, 2.7 mM KCl, 10 mM Na_2_HPO_4_, 1.8 mM KH_2_PO_4_) for 2 h at 4°C. The beads were washed four times with washing buffer (50 mM NaH_2_PO_4_, 300 mM NaCl, 20 mM imidazole), and the proteins were eluted with 2x SDS buffer, boiled for 10 min, and subjected to Western blotting with anti-GST antibody and anti-His antibody, respectively.

### Isolation and Subfractionation of Chloroplast

Leaves from 4-week-old seedlings were ground in a blender with ice-cold homogenization buffer (20 mM Tricine-KOH, pH 8.4, 450 mM sorbitol, 10 mM EDTA, pH 8.0, 10 mM NaHCO_3_, and 0.1% BSA). Homogenates were then filtered through 2-layer gauze. Stromal, thylakoid lumen, and membrane fractions were prepared as previously described with slight modifications (Hall et al., 2011). Crude chloroplasts were collected by centrifugation at 1,000 g for 10 min at 4°C. Resuspended crude chloroplasts in 1 mL of resuspension buffer (20 mM Tricine-KOH, pH 8.4, 300 mM sorbitol, 2.5 mM EDTA, and 5 mM MgCl_2_) were overlaid on Percoll step gradients [40 and 80% (v/v)] and were centrifuged at 4,000 g for 20 min. Collected chloroplasts at a chlorophyll concentration of 0.5 mg mL^–1^ were solubilized in lysis buffer (10 mM HEPES, pH 8.0) for 10 min on ice. The stromal and membrane fractions were separated by centrifugation at 18,800 g for 10 min at 4°C. The supernatant containing the stroma subfraction was collected and pellets were solubilized with 0.2% digitonin in the lysis buffer for 20 min on ice. After centrifugation at 18,800 g for 1 h at 4°C, thylakoid lumen proteins were collected from the supernatant and the membrane proteins in the pellet were resuspended in resuspension buffer.

### Isolation of Thylakoid Membrane

Thylakoid membranes were isolated from 4-week-old seedlings as described previously with minor modifications (Bhuiyan et al., 2015). Leaves were ground with a blender in ice-cold grinding buffer (330 mM sorbitol, 50 mM HEPES, pH 8.0, 1 mM MgCl_2_, 1 mM MnCl_2_, 2 mM EDTA, pH 8.0, 0.05% bovine serum albumin, and 5 mM sodium ascorbate). Homogenates were then filtered through 2-layer gauze. After centrifugation at 2,000 g for 8 min at 4°C, the pellet was resuspended in SH buffer (330 mM sorbitol, 50 mM HEPES, pH 8.0). Thylakoid membranes were isolated by centrifugation at 2,400 g for 2 min at 4°C. Pelleted membranes were resuspended in SH buffer and was used for additional analyses.

### BN/PAGE and 2D SDS/PAGE

For BN-PAGE, thylakoid membranes were resuspended in 50BTH20G buffer (50 mM BisTris, pH 7.0, and 40% glycerol) to a final concentration of 1 mg mL^–1^ chlorophyll. Thylakoid membranes were solubilized with 1% (w/v) n-dodecyl-β-D-maltoside or 4% (w/v) digitonin (Sigma-Aldrich) for 10 min on ice. After centrifugation at 10,000g for 10 min, the supernatant was mixed with 1/10 volume of BN sample buffer (100 mM BisTris-HCl, pH 7.0, 5% Serva blue G, 0.5 M 6-amino-n-caproic acid, 30% (w/v) sucrose). Then, samples were loaded onto blue native 5–13.5% polyacrylamide gradient gel. Electrophoresis was performed at 100 V in 4°C. The cathode buffer initially contained 0.01% Serva Blue G dye (Sigma-Aldrich, United States) and was replaced by buffer lacking dye after approximately half of the electrophoresis run. After electrophoresis, each lane was excised from the gel and incubated in 2x SDS buffer. Then, proteins were separated using SDS-PAGE on a 12% polyacrylamide gel. After electrophoresis, the gels were used for immunoblot analysis (Asakura et al., 2004; Lima et al., 2006).

## Results

### Conserved Residues in the C-Terminal Region Are Critical for the Structure of CYP38

**FIGURE 1:**
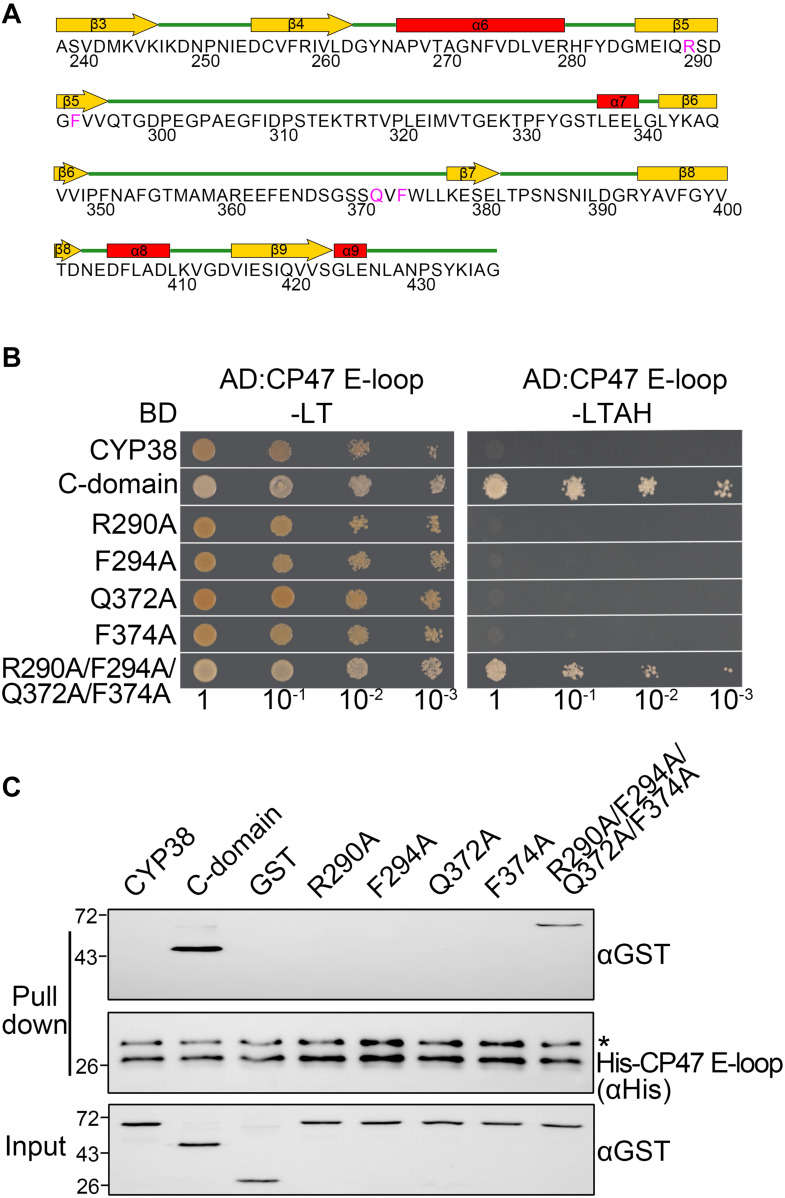
Characterization of the interaction of mutated CYP38 proteins with the CP47 E-loop. **(A)** A diagram of the secondary structure of the CYP38 C-terminal domain. The four conserved residues (R290, F294, Q372, and F374) are shown in pink. **(B)** Yeast two-hybrid analysis of the interaction between the mutated CYP38 protein and the CP47 E-loop. Yeast strain AH109 was co-transformed with the indicated combinations of constructs. Yeast cell growth on synthetic dextrose dropout medium lacking leucine and tryptophan (−LT) indicates correct co-transformation. Yeast cell growth on media lacking leucine, tryptophan, adenine, and histidine (−LTAH) indicates protein-protein interaction. **(C)** *In vitro* protein pull-down analysis of the interaction between the mutated CYP38 protein and the CP47 E-loop. Wild type and mutated CYP38 proteins expressed as GST-fusion proteins were purified by affinity chromatography. Ni-NTA agarose containing His-CP47 E-loop was incubated with approximately equal amounts of GST-fusion proteins or free GST. After washing, proteins on the beads were eluted with 2xSDS buffer and detected by immunoblotting using an anti-GST antibody and an anti-His antibody. Asterisk (*) marks unspecific bands detected by the anti-His antibody.

The residues R290, F294, Q372, and F374 in the C-terminal domain (239-437) of Arabidopsis CYP38 are highly conserved (Edvardsson et al., 2007; Figure 1A). Because CYP38 is not an active PPIase, these conserved sites should not be related to PPIase activity any more, but they could still have important impacts on the structure and function of CYP38. To test this hypothesis, we changed these residues by the site directed mutagenesis, and examined the effects of these mutations using yeast two-hybrid (YTH) and protein pull-down assays.

The C-terminal domain of CYP38, but not the mature full-length CYP38, could interact with its target, the CP47 E-loop because the N-terminal domain associates tightly with the C-terminal domain and blocks its access to the CP47 E-loop (Vasudevan et al., 2012). If a change could turn the packed CYP38 into an open configuration, then it might interact with the CP47 E-loop. Therefore, we could examine the interaction between mutated proteins and the CP47 E-loop to determine whether a mutation affects the structure of CYP38 *in vitro*.

As expected, YTH assays showed that the C-terminal domain of CYP38 (C-domain) interacted well with the CP47 E-loop, while the full length CYP38 did not (Figure 1B). When the conserved sites were individually changed to A (R290A, F294A, Q372A, and F374A), the mutated CYP38s were still unable to interact with the CP47 E-loop. By contrast, when a quadruple mutation of R290A/F294A/Q372A/F374A, referred as “the quadruple mutation” thereafter, was introduced to CYP38, a clear interaction between the mutated CYP38 and the CP47 E-loop was observed (Figure 1B and Supplementary Figure 1). Then we performed a protein pull-down experiment using His-tagged CP47 E-loop as the bait and GST–tagged mutated CYP38s as preys. The results were in agreement with the YTH experiment. CYP38 was not able to interact with the CP47 E-loop, whereas the C-domain of CYP38 could bind to this loop. The mutant CYP38 with the quadruple mutation could be captured by the CP47 E-loop, but CYP38 proteins carrying single-sited mutations did not interact with the CP47 E-loop (Figure 1C). We found that the interaction between CYP38 with the quadruple mutation and the CP47 E-loop is clearly weaker than the interaction between the C-domain and the CP47 E-loop which was observed in both YTH and protein pull-down assays (Figures 1B,C).

### The Conserved Residues Are Required for the Function of CYP38 *in planta*

**FIGURE 2:**
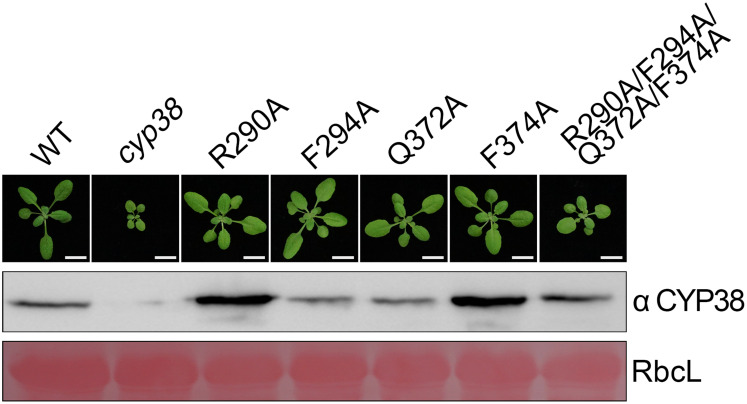
Mutagenesis of four conserved residues *in planta*. The same mutations as in the *in vitro* mutagenesis experiments were introduced into *cyp38* plants. All plants were grown for 4 weeks under continuous light (80–100 μmol m^–2^ s^–1^) after initial low light growth for 14 days as described in the section “Materials and Methods.” One representative plant for each lines. Total protein extracts were separated by SDS-PAGE and probed with an antibody against CYP38. Rubisco large subunit (RbcL) stained with Ponceau S is shown as the loading control. Bars = 1 cm.

After *in vitro* YTH and protein pull-down experiments, we further tested functional effects of those mutations *in planta*. Full-length *CYP38* cDNAs with designed mutations driven by the 35S promoter were introduced into the Arabidopsis *cyp38* mutant, which is the *cyp38-2* null allele used in a previous study (Fu et al., 2007), and the phenotypes of plants from the T_2_ generation were examined. Transgenic plants with individual changes of the four conserved residues all grew normally as wild-type plants (Figure 2). These experiments demonstrated that single mutations do not affect growth consistent with the *in vitro* experiments. Compared to wild type, transgenic plants carrying *CYP38* with the quadruple mutation (named as “quadruple mutation plants” thereafter) had a stunted growth, but less pronounced than *cyp38* (Figure 2 and Supplementary Figure 2), indicating that the quadruple mutation severely impaired, but did not fully abolish the function of CYP38 *in planta*. Immunoblotting assays to monitor protein accumulation in transgenic lines revealed that the levels of CYP38 proteins in all transgenic lines were similar to wild type. In addition, we transformed the wild-type *CYP38* cDNA into *cyp38* as a control. It rescued the mutant phenotype of *cyp38* even with a very low expression level (Supplementary Figure 3). These results indicate that the phenotypic differences of the transgenic plants can be attributed to the mutations of CYP38, but not to changes in protein expression.

### The Quadruple Mutation Does Not Affect the Interaction of the C-Domain With the N-Domain or the CP47 E-Loop

**FIGURE 3:**
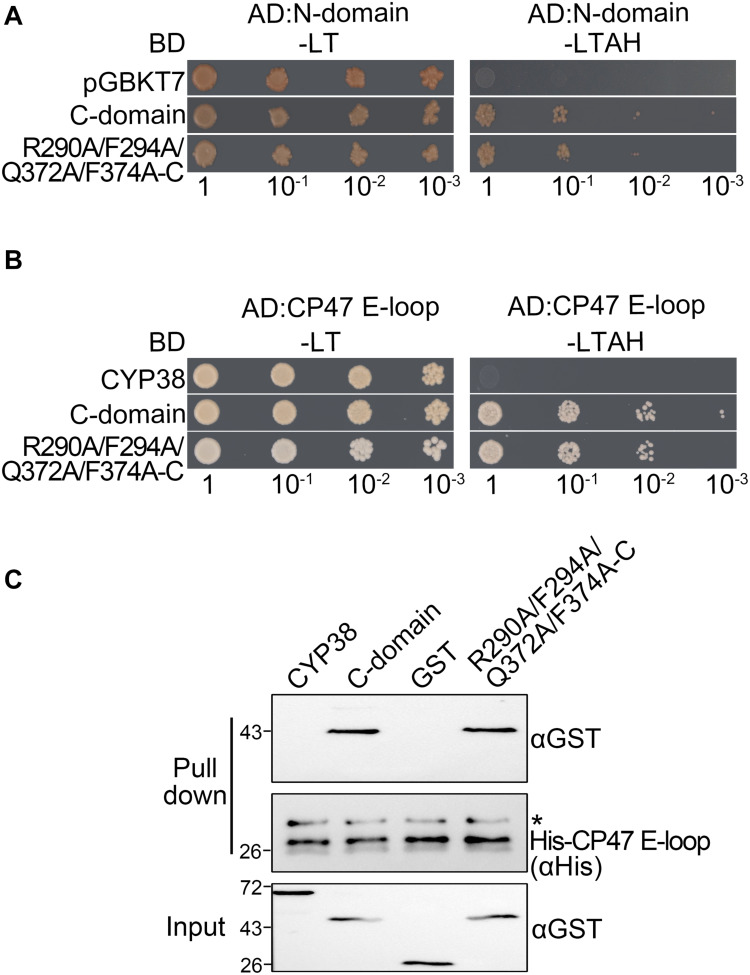
Characterization of the interaction between two C-domain isoforms with the N-domain or CP47 E-loop *in vitro*. **(A)** Yeast two-hybrid assays for interaction between the C-domain, the C-domain with the quadruple mutation (R290A/F294A/Q372A/F374A-C), and the N-domain. Yeast cells co-transformed with the indicated plasmids were selected on –LT medium, and the interaction was indicated by cell growth on −LTAH medium. **(B)** Yeast two-hybrid assays for interaction between the C-domain with the quadruple mutation and the CP47 E-loop. The legend is the same as above. **(C)** Protein pull-down assay for the interaction between the C-domain with the quadruple mutation and the CP47 E-loop. Conditions were as for Figure 1C. Asterisk (*) marks unspecific bands detected by the anti-His antibody.

When the four conserved sites were all changed to A, the mutated full length CYP38 protein was able to interact with the CP47 E-loop. This prompted us to consider that these changes might affect the interaction of the C-domain with the N-terminal domain (N-domain). To explore this possibility, we included the C-domain protein (239–437) and its mutant form with the quadruple mutation (R290A/F294A/Q372A/F374A-C) in the yeast two-hybrid assay, and tested their interaction with the N-domain protein (92–215). The result showed that the mutated C-domain, as well as the wild-type C-domain, could interact with the N-domain (Figure 3A and Supplementary Figure 1), suggesting that the quadruple mutation does not cause a significant change on the interaction between the C-domain and the N-domain. We tried to examine the same interaction using the protein pull-down assay. Unfortunately, we failed to produce the recombinant N-domain protein in spite of several attempts.

Because CYP38 with the quadruple mutation could interact with the CP47 E-loop, this raised the possibility that the mutation might influence the interaction between the C-domain and the CP47 E-loop. However, both yeast two-hybrid and protein pull-down assays showed that the mutated C-domain interacted with the CP47 E-loop as the normal C-domain (Figures 3B,C). It thus appears that the changes of these conserved residues do not obviously affect the interaction between the C-domain and the CP47 E-loop.

### The Quadruple Mutation Affects the Association of CYP38 With the Thylakoid Membrane and Impairs Its Function in PSII Assembly

**FIGURE 4:**
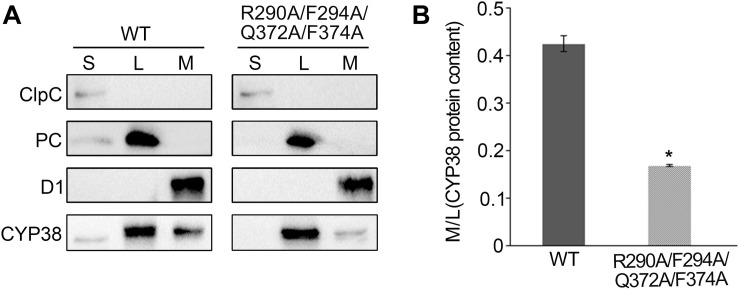
Sub-localization of CYP38 isoforms in Arabidopsis chloroplasts. **(A)** Intact chloroplasts were lysed and fractionated into stromal (S), thylakoid lumen (L), and thylakoid membrane (M), and then subjected to SDS-PAGE followed by immunoblotting. In addition to an antibody against CYP38, antibodies were used as indicated: ClpC (a stromal marker), PC (a thylakoid lumen marker), and D1 (a thylakoid membrane marker). The same volume of the fractionated stroma, thylakoid lumen and membrane fractions were loaded on the gel. **(B)** Ratio of CYP38 protein content in thylakoid membrane (M) to lumen (L). Error bars indicate SD from three biological replicates. Asterisk (*) indicates statistically significant difference between the WT and transgenic plant (R290A/F294A/Q372A/F374A) (One-way ANOVA, *p* < 0.05).

Usually, thylakoid lumen proteins exert their physiological roles via interacting with thylakoid membrane complexes, and membrane association is required for their functions (Lu, 2016). The quadruple mutation greatly affects the function of CYP38 *in vivo*, and therefore it might impair the association of CYP38 with the thylakoid membrane. This possibility was tested by fractionating isolated chloroplasts into stroma, thylakoid lumen and thylakoid membrane, and determining the sub-chloroplast localization of CYP38 by immunoblotting. The ClpC, PC, and D1 proteins were used as markers for stroma, thylakoid lumen, and thylakoid membrane, respectively. Their profiles demonstrated that the chloroplast fractionation was successful (Figure 4A). In wild type, the majority of CYP38 was present in the thylakoid lumen fraction along with a small portion on the thylakoid membrane (Figure 4A). In plants with the quadruple mutation, the ratio of the mutant CYP38 protein detected on the thylakoid membrane versa in the thylakoid lumen was significantly less than that in wild-type samples (Figures 4A,B), indicating that the quadruple mutation impaired the association of CYP38 with the thylakoid membrane.

**FIGURE 5:**
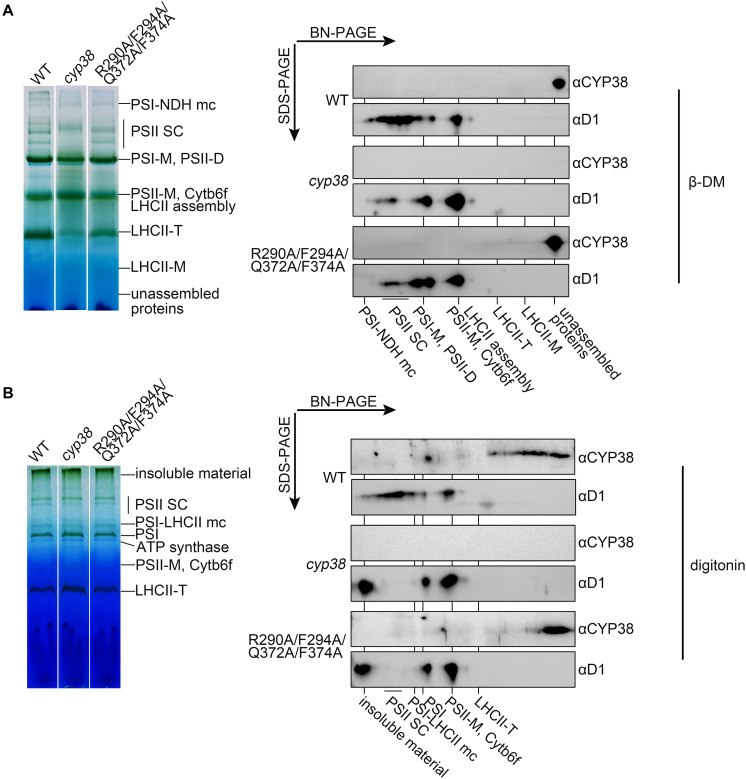
BN-PAGE analysis of thylakoid membrane complexes and association of CYP38 with membrane. Thylakoid membranes solubilized by 1% β-DM **(A)** or 4% (w/v) digitonin **(B)** were separated by BN-PAGE (15 μg chlorophyll per lane). Individual lanes were run in a second dimension by SDS-PAGE after solubilization with SDS, followed by immunoblotting with CYP38 and D1 antibodies. NDH, NAD(P)H dehydrogenase; PSII SC, PSII supercomplex; PSI-M, PSI monomer; PSII-D, PSII dimer; Cytb6f, cytochrome b6f; LHCII-T, PSII light-harvesting complex trimer; LHCII monomer, PSII light-harvesting complex monomer.

To further dissect the physiological impacts of the quadruple mutation *in planta*, blue native PAGE (BN-PAGE) analysis was used to determine how the mutation might influence PSII assembly. Purified thylakoid membranes of wild type, *cyp38*, and quadruple mutation plants were treated with N-dodecyl-β-D-maltoside (β-DM) and resolved by BN-PAGE analysis. The *cyp38* plants were almost completely devoid of PSII SCs which were obviously present in wild-type samples (Figure 5A), as reported in previous studies (Fu et al., 2007; Sirpiö et al., 2008). Several PSII supercomplex (PSII-SC) bands were missing in the BN-PAGE profile of quadruple mutation plants, which largely resembled the pattern of *cyp38* indicating a significant deficiency of PSII assembly in these plants (Figure 5A).

We further performed the second dimension SDS/PAGE after BN-PAGE, followed by immunoblotting. D1 signals were observed to overlap with PSII-SC, PSII dimer, and PSII monomer complexes in the wild-type samples, similar to the patterns shown in previous studies (Che et al., 2013; Liu and Last, 2017). In contrast, less D1 signals in PSII-SC and more in PSII dimer, and PSII monomer were detected in the mutant samples, indicating that CYP38 with the quadruple mutation cannot normally function in PSII assembly (Figure 5A). When blotted with antibody against CYP38, it showed that CYP38 predominantly co-migrated with unassembled free proteins in both the wild-type and mutant samples, probably because β-DM is a relatively strong detergent, which could break the weak interaction between CYP38 and the thylakoid membrane. Therefore we replaced β-DM with a milder detergent digitonin according to previous studies (Järvi et al., 2011; Myouga et al., 2018). The BN-PAGE profiles with digitonin were different from that with β-DM (Figure 5B). Once again, D1 signals were observed at the positions corresponding to PSII-SC, PSII dimer, and PSII monomer complexes in the wild type samples. On the other hand, a large amount of D1 signals were detected in the insoluble material, PSII dimer, and PSII monomer fraction in the mutant samples. Wild-type CYP38 was found to co-migrate with a series of thylakoid membrane components with molecular weights smaller than the LCHII-T complex and of still unknown composition. In the quadruple mutation plants, most of the mutated CYP38 protein was either associated with small-sized thylakoid membrane complexes or present in the unassembled free protein fraction, strikingly distinct from the pattern of wild type (Figure 5B).

## Discussion and Conclusion

Proper assembly and maintenance of PSII is essential for photosynthesis. A large number of auxiliary proteins, including thylakoid lumen immunophilins, have been identified as protein factors involved in PSII assembly and repair (Nixon et al., 2010; Nickelsen and Rengstl, 2013; Lu, 2016). So far, studies on these protein factors were mainly restricted to the mutant identification and characterization of its defects, leaving their working mechanisms elusive.

Arabidopsis CYP38, a PSII assembly factor in the thylakoid lumen, contains a PPIase like domain in its C-terminal region that is inactive PPIase (Shapiguzov et al., 2006; Vasudevan et al., 2012), suggesting that CYP38 could have acquired a novel function in chloroplasts. The C-terminal domain, but not the N-terminal domain, interacts with a number of potential target proteins (Vasudevan et al., 2012), implying that the C-terminal domain is still the functional domain, while the N-terminus acts as a regulatory domain.

We propose that CYP38 is initially present in a packed state in the thylakoid lumen and that its function is governed by the chloroplast energy status in chloroplasts. After the acidification of the thylakoid lumen through light-induced photosynthetic electron flow, CYP38 docks onto the thylakoid membrane. The packed CYP38 changes conformation into an open state induced by the lower pH. Then, the C-terminal domain is no longer inhibited by the N-terminus, and binds its targets, such as the CP47 E-loop, to assist the proper assembly of different forms of PSII complexes. After CYP38 completes its assembly function, it will detach from its substrate and form a packed state again by interacting with the N-terminal bundle. Finally, CYP38 will dissociate from the thylakoid membrane and move back to the thylakoid lumen.

Due to the presence of a PPIase like structure in CYP38, it is possible that CYP38 preferentially binds peptides similar to substrates of PPIases. We noticed that there are a number of proline residues in the CP47 E-loop (Bricker and Frankel, 2002 and Supplementary Figure 4), making it a favorite substrate of CYP38. The remaining conserved sites of CYP38 may not be related to PPIase activity anymore, but still crucial for the novel function of CYP38 in chloroplasts. Possibly, CYP38 could act as a chaperone-like protein to interact with PSII subunits, and the conserved sites could be involved in maintaining target proteins in the right position or right configuration during the dynamic and rapid process of PSII assembly and repair.

Conserved residues in the C-terminal region (R290, F294, Q372, and F374) could be important for the protein structure and function, even if they are not related to PPIase activity. Each single mutation at these four sites did not affect the *in vitro* performance of CYP38 (Figure 1), showing that single mutations would have a very small impact on the structure of CYP38 without interfering with its function. The quadruple mutation significantly changed the behavior of CYP38 *in vitro* (Figure 1), suggesting that these combined alterations result in a change in structure of CYP38 and impair its function. The results of gene transformation experiments *in planta* are consistent with the *in vitro* yeast two-hybrid and protein pull-down assays. Single mutations did not impair the function of CYP38, but the quadruple mutation significantly impaired CYP38 as shown by its failure to fully complement the growth defects of *cyp38* mutant plants (Figure 2 and Supplementary Figure 2).

R290 and F294 are located at the β5 sheet, while Q372 and F374 are just in front of the β7 sheet, and both sheets could be very important for the structural integrity of the C-terminal domain (Figure 1A). We compared the CYP38 containing the quadruple mutation with the wild type CYP38 using the I-TASSER prediction (Yang and Zhang, 2015), and found that a substantial change occurs in the β7 and β8 region of the mutant protein (Supplementary Figure 5). The structural change caused by the quadruple mutation should be further confirmed by actual structural analysis by CD, X-ray, or Cryo-EM approaches.

Because CYP38 with the quadruple mutation interacts with the CP47 E-loop, we assume that the intramolecular N-C interaction in the mutated CYP38 is weaker than that in wild type. Therefore the CP47 E-loop would prevail in the competition with the N-domain of CYP38, and bind the full length mature CYP38 protein. However, we found that the mutant C-domain half protein still interacted with the N-terminal half protein in YTH assays, similar as its wild-type counterpart (Figure 3A). It is possible that the change in the intramolecular interaction inside the mutated CYP38 is too weak to be detected in the *in vitro* assay.

The membrane association assay showed that CYP38 with the quadruple mutation associated less with thylakoid membrane complexes than the wild-type CYP38 (Figure 4). BN-PAGE analysis indicated that the mutated CYP38 is only associated with a few small sized complexes, unlike wild-type CYP38 bound to a series of thylakoid membrane assembly intermediates (Figure 5B). We propose that the quadruple mutation impairs the interaction between CYP38 and its target substrates, and further compromises its chaperone-like assembly function in chloroplasts. However, both YTH and pull-down assays showed that the C-domain carrying the quadruple mutation, just like the wild-type C-domain, could bind the CP47 E-loop indicating that the mutated C-domain is still able to bind its target proteins (Figures 3B,C). The discrepancy between the results from *in planta* and *in vitro* experiments could be explained that the *in vitro* experiments were not sensitive enough to reflect processes occurring *in planta*.

In conclusion, we consider that the conserved amino acids examined in this study are critical for CYP38 to maintain its dynamic intramolecular N-C interaction and the ability to bind its target proteins. Mutations in those sites could weaken the N-C interaction and diminish its regulatory mechanism in response to changing light conditions. The mutated CYP38 could still recognize and bind its target proteins, but its alteration would affect its function *in vivo*.

We noticed that most of the thylakoid lumen immunophilins, except CYP20-2 and FKBP13, are divergent and have lost most of the conserved residues required for PPIase activity, similar to CYP38 (He et al., 2004; Lima et al., 2006; Edvardsson et al., 2007). The remaining conserved sites in those divergent immunophilins could be also important for their function as in the case of CYP38. In addition to CYP38, FKBP20-2 is another important immunophilin in the thylakoid lumen, and *fkbp20-2* mutant plants showed a stunted growth phenotype as well (Lima et al., 2006). It is worthwhile to perform a similar research on FKBP20-2, to examine the effects of these conserved sites on its structure and function. It is possible that all inactive PPIase immunophilins could function through a similar mechanism in the thylakoid lumen.

## Data Availability Statement

The original contributions presented in the study are included in the article/Supplementary Material, further inquiries can be directed to the corresponding author/s.

## Author Contributions

AF, YW, MX, and LS designed the research. LS, LD, JWe, XZ, WZ, and JWa performed the research. LS, YW, MX, and AF analyzed the data. LS, YW, and AF wrote the manuscript with contribution and approval from all authors.

## Conflict of Interest

The authors declare that the research was conducted in the absence of any commercial or financial relationships that could be construed as a potential conflict of interest.
