# Supplementary material for: Conserved Residues in the C-Terminal Domain Affect the Structure and Function of CYP38 in Arabidopsis
Source: Front Plant Sci. 2021 Feb 25;12:630644. doi: 10.3389/fpls.2021.630644 (PMC7959726; doi:10.3389/fpls.2021.630644)
Supplement: Supplementary file 1 [file Data_Sheet_1.pdf]

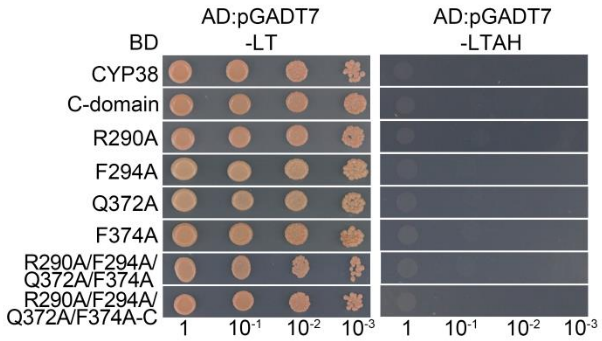

Supplementary Figure 1. Yeast two-hybrid assay between the mutated CYP38 proteins and pGADT7.

Yeast two-hybrid analysis of the interaction between the mutated CYP38 proteins and pGADT7. Yeast strain AH109 was co-transformed with the indicated combinations of constructs. The transformants were grown on the synthetic dextrose dropout medium lacking leucine and tryptophan (-LT) or on the media lacking leucine, tryptophan, adenine, and histidine (-LTAH)

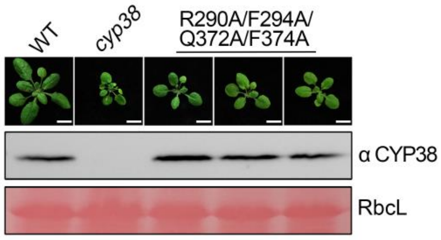

Supplementary Figure 2. Phenotypes of Arabidopsis WT, *cyp38* and transgenic lines (R290A/F294A/Q372A/F374A).

All plants were grown for 4 weeks under continuous light (80-100 μmol m^{-2} s^{-1}) after initial low light growth for 14-days. Total protein extracts were separated by SDS-PAGE and probed with the CYP38 antibody. The large subunit of Rubisco (RbcL) served as the loading control. Bars = 1cm.

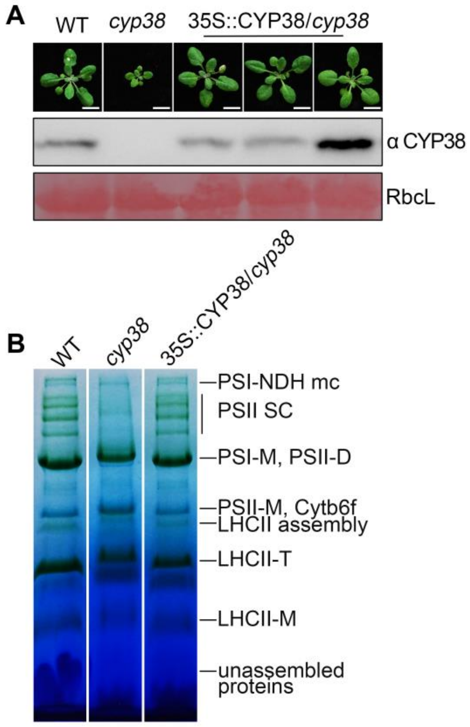

Supplementary Figure 3. Complementation of cyp38 with wild type CYP38.
(A) Phenotypes of the WT, *cyp38* and three independent complemented lines overexpressing *CYP38* under the 35S promoter in *cyp38* (35S::CYP38/*cyp38)*. Total protein extracts were separated by SDS-PAGE and probed with the CYP38 antibody. The large subunit of Rubisco (RbcL) served as the loading control. Bars = 1cm; **(B)** BN-PAGE analysis of thylakoid membrane complexes from WT, cyp38, and complemented line after 4 weeks of growth. Thylakoid membranes corresponding to equal amounts of chlorophyll (15µg) were solubilized with 1% β-DM and separated by BN-PAGE.

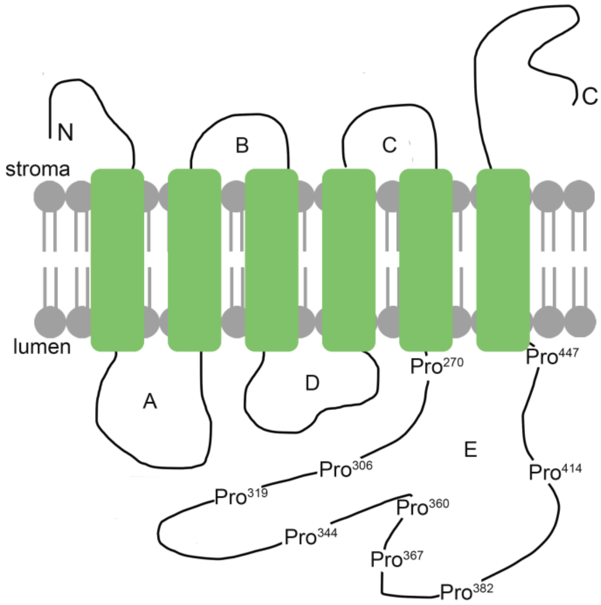

Supplementary Figure 4. A schematic structure of the CP47 protein.
Six transmembrane helices of the CP47 proteins, connected by stromal and luminal loops. A-E indicated the extrinsic loop regions. The positions of the N and C termini are indicated. The approximate locations of the proline residues in the CP47 E-loop are addressed.

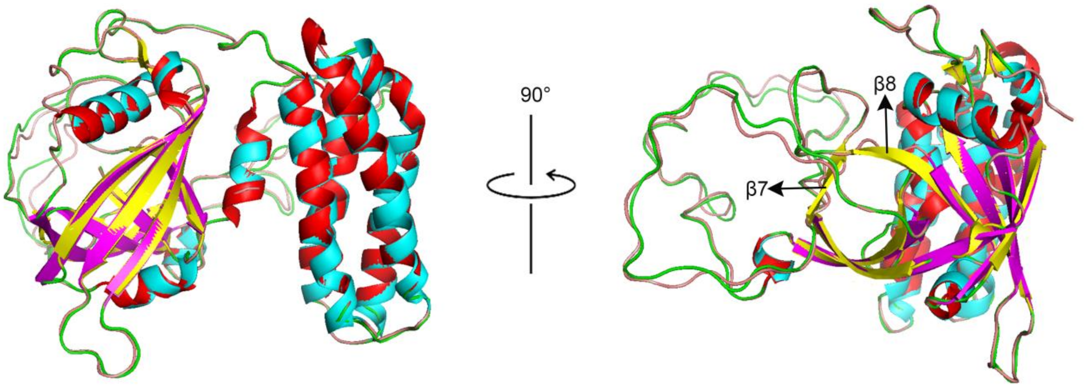

Supplementary Figure 5. Alignment of the structures of CYP38 and its mutant containing the quadruple mutation in the ribbon model by I-TASSER. Overall structure of CYP38 (92 to 437) with α-helices shown in red, β-strands in yellow, and loops in green (PDB ID: 3RFY). Overall structure of the mutated protein (92 to 437) predicted by I-TASSER with α-helices shown in blue, β-strands in purple, and loops in pink. The mutated protein shows a loop structure at the original β7 and β8 sheet region of CYP38. The structure figures were prepared using PyMOL.

Supplementary Table 1: Primers used in this study

Primer Name	Sequences 5'-3'	Used for
R290A-F	GCATGGAGATCCAGGCATCT GATGGATTTGTG	Site-directed Mutagenesis
R290A-R	CACAAATCCATCAGATGCCT GGATCTCCATGC	Site-directed Mutagenesis
F294A-F	GAGATCTGATGGAGCTGTGG TACAAACG	Site-directed Mutagenesis
F290A-R	CGTTTGTACCACAGCTCCATC AGATCTC	Site-directed Mutagenesis
Q372A-F	GACTCAGGATCAAGCGCAGT GTTTTGGCTGC	Site-directed Mutagenesis
Q372A-R	GCAGCCAAAACACTGCGCTT GATCCTGAGTC	Site-directed Mutagenesis
F374A-F	GGATCAAGCCAAGTGGCTTG GCTGCTAAAAG	Site-directed Mutagenesis
F374A-R	CTTTTAGCAGCCAAGCCACT TGGCTTGATCC	Site-directed Mutagenesis
mature-CYP38-F	ACGCCTCGAGATGGCGGCGG CGTTTGCCTCTCTTC	cloning
mature-CYP38-R	GCCGAGCTCTTAACCGGCGA TTTTGTAACTCGGGTTAGCG	cloning
GST-CYP38-F	CCCCTTGGATCCTCAGTGTT GATCTCCGGTCCTC	cloning
GST-CYP38-R	CCCTCGAGTTAACCGGCGAT TTTGTAACTC	cloning
YTH-CYP38-F	GGGGAATTGTCAGTGTTGAT CTCCG G	cloning
YTH-CYP38-R	GAGCTCGAGACCGGCGATTT TGTAAC	cloning
Full-CYP38-F	ACGCGTCGACATGGCGGCGG CGTTTGCCTCTCTTC	cloning
Full-CYP38-R	GCCGAGCTCTTAACCGGCGA TTTTGTAACTCGGGTTAGCG	cloning
CP47 E-loop-F	CCGGAATTCGAATTATTTGGT CCTACTCGTTATCAATGGG	cloning
CP47 E-loop-R	CCGCTCGAGCTACCAACCTC TTGGGCTGCTACG	cloning
